# Perception of stochastic envelopes by normal-hearing and cochlear-implant listeners

**DOI:** 10.1016/j.heares.2015.12.013

**Published:** 2016-03

**Authors:** Philip A. Gomersall, Richard E. Turner, David M. Baguley, John M. Deeks, Hedwig E. Gockel, Robert P. Carlyon

**Affiliations:** a MRC Cognition & Brain Sciences Unit, 15 Chaucer Rd., Cambridge, England, United Kingdom; b Addenbrookes NHS Trust, Hills Rd., Cambridge, England, United Kingdom; c Anglia Ruskin University, East Rd, Cambridge, England, United Kingdom; d Dept. Engineering, University of Cambridge, Trumpington Street, Cambridge, England, United Kingdom

**Keywords:** Sound textures, Envelope discrimination, Cochlear implants, CI, Cochlear Implant, NH, Normal Hearing, MDS, Multidimensional Scaling, SMD, Statistical Modulation Depth, rms, root-mean-square, DL, Discrimination Limen, ANOVA, Analysis of Variance

## Abstract

We assessed auditory sensitivity to three classes of temporal-envelope statistics (modulation depth, modulation rate, and comodulation) that are important for the perception of ‘sound textures’. The textures were generated by a probabilistic model that prescribes the temporal statistics of a selected number of modulation envelopes, superimposed onto noise carriers. Discrimination thresholds were measured for normal-hearing (NH) listeners and users of a MED-EL pulsar cochlear implant (CI), for separate manipulations of the average rate and modulation depth of the envelope in each frequency band of the stimulus, and of the co-modulation between bands. Normal-hearing (NH) listeners' discrimination of envelope rate was similar for baseline modulation rates of 5 and 34 Hz, and much poorer than previously reported for sinusoidally amplitude-modulated sounds. In contrast, discrimination of model parameters that controlled modulation depth was poorer at the lower baseline rate, consistent with the idea that, at the lower rate, subjects get fewer ‘looks’ at the relevant information when comparing stimuli differing in modulation depth. NH listeners could discriminate differences in co-modulation across bands; a multidimensional scaling study revealed that this was likely due to genuine across-frequency processing, rather than within-channel cues. CI users' discrimination performance was worse overall than for NH listeners, but showed a similar dependence on stimulus parameters.

## Highlights

• We measure discrimination thresholds for input parameters of a stochastic sound texture generator.
• CochIear Implant listeners performed less well than Normal Hearing listeners.
• Discrimination of parameters controlling modulation depth in a channel was better at higher overall modulation rates.
• Rate discrimination is poorer for our complex stochastic modulation envelopes than ‘deterministic’ stimuli.
• Changes in across-modulator interdependence may be derived by summing the outputs across multiple auditory filters.

## 1 Introduction

In recent years there has been an emerging interest in the perception of a class of stimuli known as “sound textures”. These include familiar environmental sounds such as fire, wind, rain and running water. Research from two groups (McDermott, 2009, Turner and Sahani, 2010, McDermott and Simoncelli, 2011, McDermott et al., 2013) indicates that listeners process and identify sound textures using information derived from the statistics of the envelopes in each frequency band, and from the relative amplitudes of, and correlations between them. Evidence for statistics-based perception comes from the fact that models that use a small number of stochastic parameters can generate sounds that can be readily identified as originating from different categories (McDermott, 2009, Turner and Sahani, 2010, McDermott and Simoncelli, 2011).

A large number of studies have investigated the detection of differences in stimulus envelope parameters by normal-hearing listeners. These include the detection of both first- and second-order amplitude modulation, the discrimination of changes in modulation rate, and the detection of differences in comodulation between pairs of narrowband carriers (Füllgrabe and Lorenzi, 2003, Grant et al., 1998, Lee, 1994, Moore and Emmerich, 1990, Richards, 1987). The vast majority of those studies have used deterministic modulators, and/or have imposed the modulation on either a single carrier or on a small number of carriers. This differs from many environmental sounds, which contain stochastic modulation in multiple contiguous frequency bands. In contrast, research on sound-texture perception has either required listeners to make categorisation judgements (McDermott and Simoncelli, 2011, Turner and Sahani, 2010), or, where discrimination experiments have been performed, have involved the simultaneous manipulation of more than one stimulus parameter (McDermott et al., 2013). In an attempt to bridge this gap by providing basic psychophysical measures of sensitivity to the envelope parameters important for the perception of sound textures, the present study measured listeners' sensitivity to differences in the individual parameters of one generative model, previously described by Turner and Sahani (2010). We then compare the results of both discrimination and multi-dimensional scaling experiments to the predictions of a simple auditory model, in order to gain insight into the auditory cues that listeners use when distinguishing between sound textures. For example, we find that increasing the co-modulation between the envelopes in different frequency bands increases the modulation depth both in individual auditory filter outputs and in the summed envelopes of all auditory filters that respond to the sound, but that listeners are either primarily or exclusively sensitive to the summed (across-channel) cue. We also demonstrate a formal mathematical relationship between the summed-channel cue and one in which each channel's envelope is correlated with every other, and discuss the implications of this relationship for the interpretation of results from other paradigms, such as the discrimination of correlation applied to widely-spaced narrowband carriers, and to the co-modulation detection difference (CDD: Cohen and Schubert, 1987, McFadden, 1987).

An understanding of the perception of the envelopes in each frequency region of broadband sounds, and of the correlation between those envelopes, is arguably of even more importance to the study of hearing by cochlear implant (CI) users. The speech-processing strategies implemented in all contemporary CIs discard the temporal fine structure in either all or the majority of frequency channels (McDermott et al., 1992, Wilson et al., 1991) leaving the listener to rely primarily on the channel envelopes to distinguish between the sounds encountered in everyday life. As is the case for normal acoustic hearing, research on envelope perception by CI users has typically used deterministic envelopes applied either to a single carrier or to a small number of carriers (Cazals et al., 1994, Chatterjee and Oba, 2004, Chatterjee and Robert, 2001, Chatterjee, 2003, Fu, 2002, Lorenzi et al., 1998, Lorenzi et al., 1997, Richardson et al., 1998, Shannon, 1992, Won et al., 2011). We are not aware of any psychophysical investigation into sound-texture identification by CI users. We therefore repeated a subset of the experiments with CI listeners, as a first step towards an understanding of how this population perceive differences between sound textures.

Our investigation of stochastic envelope processing by CI users is also relevant to a long-term clinical goal of the study, related to the alleviation of tinnitus in CI users by the presentation of competing sounds (“sound therapy”). The environmental sounds used in this form of therapy correspond very closely to those used in the study of sound textures and that are effectively reproduced by generative models. Although the evidence for the overall effectiveness of sound therapy is equivocal (Hobson et al., 2010), exposure to low-level auditory textures can alleviate sleep handicap in tinnitus sufferers with acoustic hearing (Handscomb, 2006). Unfortunately, the benefit of such stimuli to CI users with tinnitus has not been established, and, in a preliminary stage of a previous study (Carlyon et al., 2010), CI users reported that the environmental sounds used to alleviate tinnitus in acoustic hearing sounded similar to each other. Indeed, many of the fast fluctuations present in these stimuli were degraded by the processing of the CI. By using a generative model, rather than simply selecting from a range of available sounds, it may be possible to produce stimuli that evoke a wider range of percepts, and, potentially, lead to a method where an individual CI patient can select an effective sound by varying a small number of parameters. Important pre-requisites to this goal include an understanding of listeners' sensitivity to changes in the parameters of a generative model, and of how these acoustic changes are processed by the electrically stimulated auditory system.

## 2 Generative model

The stimuli were created using a statistical audio texture model (Turner and Sahani, 2010). The model generates signals by summing a set of quickly varying band-limited noise carriers that have slowly-varying modulation imposed upon them by a set of stochastic modulators. The statistics of the signal are controlled by a set of parameters that intuitively correspond to the bandwidths and centre frequencies of the narrow-band noise carriers, the modulation-depth (or sparsity) and the rate of envelope fluctuations, and the dependencies between the modulators.

The full statistical audio texture model contains a large number of parameters that control fine details of the statistical structure of the generated sounds. In the original work, these parameters allowed the model to match the statistics of target textures, such as running water, crackling fire, or howling wind. Here the goal is rather different since we are interested in exploring perceptual sensitivity to the three main classes of envelope statistics, namely the modulation depth, modulation rate, and the dependencies between the modulators (comodulation). For this reason, we use a simpler version of the model with fewer parameters that is nevertheless able to produce a range of textural sounds that were subjectively identified by the authors to sound fairly naturalistic, and that result in similar range of statistics as natural sounds at the output of an auditory model (see Section 6.2, also examples available at http://www.mrc-cbu.cam.ac.uk/wp-content/uploads/2015/11/Wind.wav, http://www.mrc-cbu.cam.ac.uk/wp-content/uploads/2015/11/Water.wav, http://www.mrc-cbu.cam.ac.uk/wp-content/uploads/2015/11/Rain.wav). We focus on sensitivity to temporal fluctuations rather than to differences in spectral shape, which are already reasonably well accounted for by existing auditory models. Therefore all stimuli had the same long-term spectrum.

**Fig. 1:**
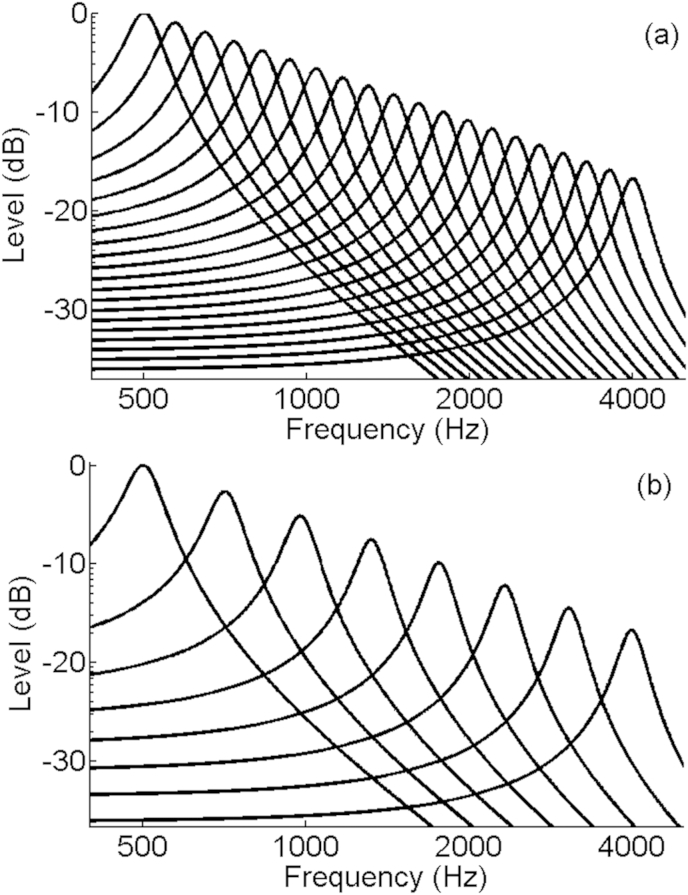
The filter shapes used to generate the narrowband noise carriers, in the (a) 20-carrier and (b) 8 carrier conditions.

As the focus of this paper is on the perceptual sensitivity to the statistical properties of the modulators, the statistics of the narrow-band carriers were fixed throughout. Specifically, the carriers were produced by filtering Gaussian noise through band-limited filters (defined by second order autoregressive functions), with centre frequencies equally spread in log space between 500 and 4000 Hz. The bandwidths and centre frequencies used for the 20-carrier stimuli employed in most of our experiments are shown in Table 1a, and the filters imposed on each carrier are plotted in Fig. 1a.

The modulators were generated in three stages. The first stage produces low-pass Gaussian noise, which is both smooth and slowly varying, and the second stage converts this into a positive modulator. The third stage introduces dependencies between the modulators in different frequency channels.

**Fig. 2:**
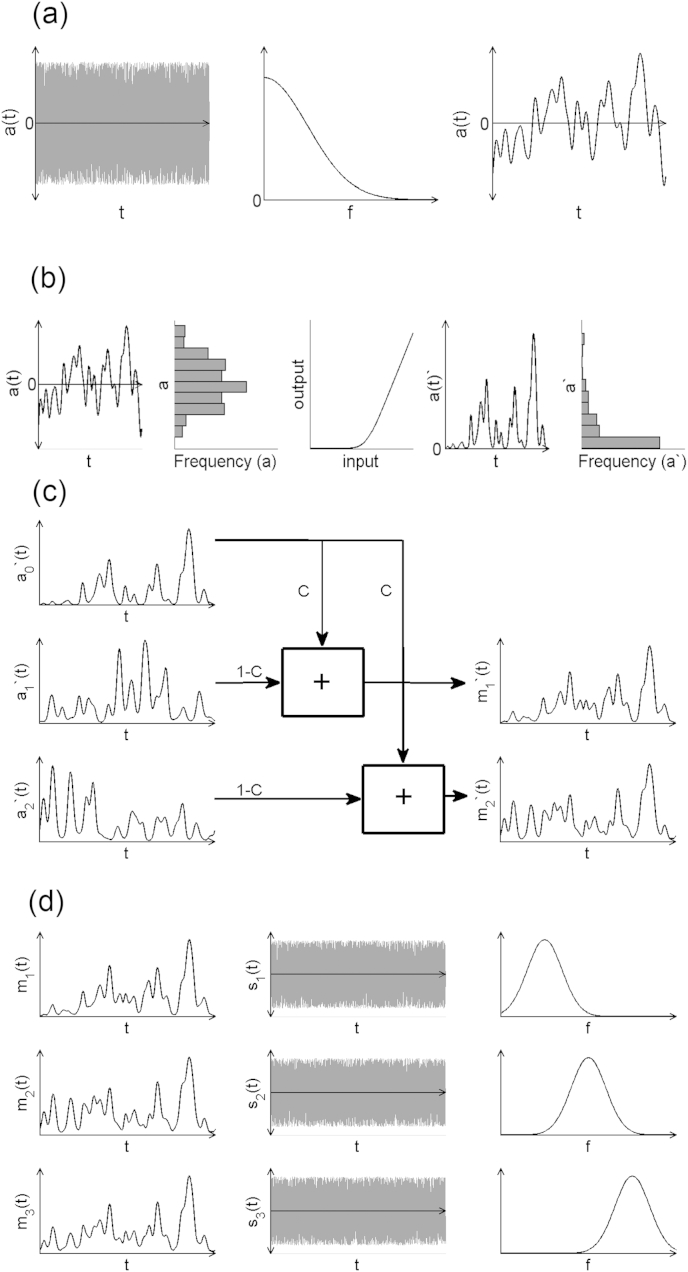
Schematic representation of stimulus generation method. (a) A broadband noise is low-pass filtered using a Gaussian filter centred on frequency = 0, resulting in a fluctuating waveform with characteristic timescale of fluctuation = 1/*f*_*0*_. (b) The output of (a) is passed through a positive non-linearity. This results in a highly skewed distribution of amplitudes, which better characterises the fluctuations in amplitude of natural sounds. (c) Multiple envelopes are independently generated, each of which is used to modulate a narrow band noise carrier. The noise carriers are equally spread in log space across the frequency range 500–4000 Hz (see Fig. 1). The amount of co-modulation is controlled by linear addition of a private modulator weighted by (1-C), and a shared modulator weighted by C, where C varies from 1 (perfect co-modulation) to 0 (independent modulators imposed on each carrier) (d) Envelopes (lefthand column) are superimposed on bandlimited noise carriers (middle column) whose spectra are shown schematically (righthand column).

In more detail, in the first stage (Fig. 2a) the low-pass Gaussian noise is produced using a filter that has a Gaussian shape,

(1) $$W\left(f\right)=Ae^{-f^{2}/\left(2f_{o}^{2}\right)}$$

The filter is centred on 0 Hz and has a parameter *f*_*0*_ that controls the width of the low-pass filter and therefore the rate of fluctuation in the envelope (henceforth referred to as modulation rate). In the time domain *f*_***0***_ controls the characteristic time-scale of the envelope *t*_*0*_ = 1/*f*_*0*_. The second parameter A sets the gain of the output of the filter. In the second stage (Fig. 2b), the low-pass Gaussian noise is converted into a positive modulator by passing it through a positive instantaneous non-linearity (equation (2)),

(2) $$a\left(t\right)=log\left(1+e^{x\left(t\right)-B}\right)\text{,}\;B>0$$

This transformation is a soft version of the threshold linear function: when the input noise, *x(t)*, is much smaller than the off-set parameter, B, the modulator is close to zero. In contrast, when the input noise is much greater than the offset parameter and also much greater than one, the modulator is a linear function of the input, *a(t)* = *x(t)*-*B*. In this way the offset parameter controls the proportion of the modulator that is close to zero and, together with the gain parameter A, it determines the statistical properties of the modulation including the mean value and the variance. Both of these parameters affect the modulation depth (or “sparsity”) of the envelope; their effect on other envelope properties such as skew and kurtosis is discussed in Section 6.4. In our implementation, the values of A, B, and *f*_*0*_ are held constant for all filters for a given sound.

The final statistic that will be manipulated is the degree of correlation between the modulators in different frequency bands (Fig. 2c). This is controlled by mixing a private modulator in each sub-band (*a*_*n*_(*t*)) with a shared modulator (*a*_0_(*t*)),

(3) $$m_{n}\left(t\right)=C\;a_{0}\left(t\right)+\left(1-C\right)a_{n}\left(t\right)\text{.}$$

When the comodulation parameter *C* is equal to zero, the modulation in each frequency band is equal to a private modulator and therefore independent of the other channels. In contrast, when the comodulation parameter is equal to one, the modulation in each frequency band is equal to the shared modulator. The modulators are then imposed on bandpass-limited noise carriers (each carrier is multiplied by the corresponding modulation envelope) (Fig. 2d).

**Fig. 3:**
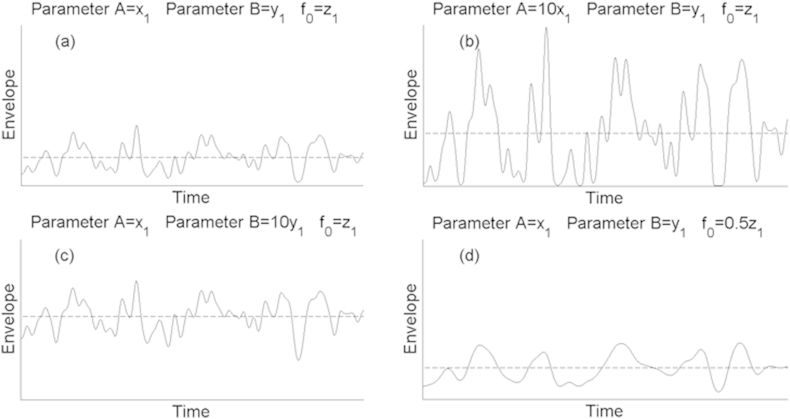
A diagrammatic representation of the influence that parameters A, B, and *f*_*0*_ have on the envelope characteristics of a single channel. In all panels the x-axis is time and the y-axis is amplitude. In each case the broken line represents the mean value for the envelope. Comparison of panels (a) and (b) demonstrates how changes in parameter A correspond primarily to a change in the variance of the raw waveform, i.e. a change in the typical deviation from its mean value, with only a slight increase in this mean value as the parameter value is increased. A change in parameter B of a similar order of magnitude results in a larger shift in the envelope mean value as seen by comparing panels (a) and (c). The effect of changing the stochastic modulation rate (*f*_*0*_) can be seen by comparing panels (a) with panel (d).

In summary, we use a version of the statistical audio texture model in which the statistics of the modulators in each frequency band are identical. The modulator statistics are completely described using four input parameters: A, B, *f*_***0,***_ and C (comodulation). Parameters A and B control the modulation depth or sparsity of the signal, *f*_*0*_ controls the modulation time-scale, and C controls the dependencies between the modulators. Parts a through d of Fig. 3 illustrate the effects of changing A, B, and *f*_*0*_ on the envelope applied to a single channel. In our experiments all stimuli were presented at the same overall root-mean-square (rms) level, which had the effect of reducing differences in the overall amplitudes of the envelopes.

## 3 Auditory model and summary statistics

The sound textures used here and elsewhere (McDermott and Simoncelli, 2011, McDermott et al., 2013, Turner and Sahani, 2010) differ from those used in the majority of psychoacoustical studies of modulation perception in at least two respects. First, because the stimuli are stochastic, the depth of modulation will be different between ‘cycles’, where a cycle is described as the portion of the envelope waveform sitting between two local maxima or minima. As a consequence the standard definition of modulation depth cannot be applied. We use the metric proposed by Turner (2010): the *statistical modulation depth (*SMD*)*, which is the ratio of the standard deviation of the waveform envelope to the mean envelope value (σ/μ). Second, and perhaps more importantly, the modulators are applied to spectral bands that are contiguous and that have some degree of overlap (Fig. 1a; Table 1a). Hence the modulation applied to one noise carrier can affect the waveform at the output of an auditory filter centred on the carrier frequency of an adjacent band. For example, when all bands are co-modulated then the SMD in individual auditory filter outputs will tend to be greater than when adjacent frequency regions of the stimulus are modulated independently.

To distinguish between across- and within-channel processing, we compared the results of our experiments to the output of an auditory model. Stimuli were processed by a 24-channel fourth-order gammatone filterbank, and the envelope of each filter output was extracted (half-wave rectified and low-pass filtered). The resulting envelopes were time-aligned by delaying each channel output such that the impulse response in each channel was centred on the same time point, using the align function of MATLAB's gammatone filterbank implementation (Cooke, 1991). We then calculated three metrics: (i) *Within-channel SMD*, defined as the SMD averaged across channels, (ii) *Across-channel SMD*, calculated by summing the envelope at the output of each auditory filter and measuring the SMD of this summed output, and (iii) *Across-channel correlation,* produced by calculating the correlation between each auditory filter envelope and every other, and taking the mean of these values. A formal relationship between these three parameters, not restricted to the particular set of stimuli used here, is described in the Appendix. In general terms, for any set of sounds, changes in one parameter will typically affect at least one of the other two. For example, increasing the within-channel SMD whilst maintaining the same correlations between channels increases the across-channel SMD. It is also possible for two stimuli to produce the same within-channel SMD, but for one to produce both a larger cross-channel correlation and a larger across-channel SMD than the other. The experimental question is then whether these two stimuli sound alike, and hence whether or not within-channel cues dominate perception.

Another method that we used to evaluate the relative importance of across- and within-channel cues was to repeat some of the experiments with a subset of the listeners and with 8-channel stimuli. These stimuli, whose characteristics are shown in Table 1b and Fig. 1b, consisted of carriers that were more sparsely distributed than the 20-carrier stimuli used in the majority of experiments. This would have reduced the extent to which adjacent carriers interacted in the outputs of individual auditory filters. If the level and/or pattern of performance depended strongly on these interactions then it should differ between the eight- and twenty-channel stimuli.

## 4 .Discrimination experiments

### 4.1 Methods

#### 4.1.1 Normal-hearing listeners

Stimuli were generated using a MATLAB implementation of the statistical audio texture model described above. A 20-s waveform was generated for each combination of input values. For each presentation a 1000-ms sample was randomly chosen from the longer waveform and converted into a 32-bit resolution wave file with a 16-kHz sampling frequency. Stimuli were up-sampled to 44.1 kHz, D/A converted with a sound card (ASUS Xonar Essence STX, 24-bit, 44.1 kHz sampling frequency) and attenuated using a programmable attenuator (TDT PA4). They were then diotically presented, via a headphone buffer (TDT HB6), over Sennheiser HD 650 headphones to a listener seated in a double-walled sound-treated booth. Prior to testing, the presentation level was adjusted to be audible and comfortable for a normal hearing listener. This level was equivalent to 56 dB SPL. All stimuli had equivalent root-mean-square values.

Five listeners with audiometric thresholds of 10 dB HL or better at octave frequencies between 500 and 4000 Hz took part. One listener (S1) was the first author; all other listeners received payment for participation and gave written informed consent prior to each session.

Each trial consisted of three presentations, containing one combination of test (T) and standard (S) stimuli from the following sequential ordering: TTS, TST, STS, and SST. A combination of fixed input values was used in order to generate standard stimuli; test stimuli were generated by varying one selected input parameter for the model over a range of values, whilst holding the other parameters at a constant value. Each listener received written instructions explaining that the task was to identify the ‘most different’ of the three sounds, constrained to the second and third interval. Note that different exemplars were presented in all three intervals, but that two stimuli shared the same long-term statistics. Hence the task is a modification of the traditional three-interval two-alternative task, which we refer to as “oddest man out”; this paradigm has been used previously to investigate perceptual differences between sound textures (McDermott et al., 2013). After each trial listeners indicated their choice of interval for the oddest-man-out stimulus using a graphical user interface (GUI). Visual feedback was then provided on the basis of the input value for the test parameter. Every experiment was balanced across the four possible sequential orderings of the standard and test stimuli. The sequence in which the stimulus combinations were presented was randomised by generating all combinations and randomly picking without replacement. This procedure was used for all discrimination experiments reported here.

Within a session the discrimination task for a particular parameter was performed once at a higher modulation rate (*f*_*0*_ = 34 Hz) and once at a lower modulation rate (*f*_*0*_ = 5 Hz). All four parameters (A, B, C, and *f*_*0*_) were tested at both rates leading to eight conditions in total. For the co-modulation parameter the standard stimulus always had a co-modulation (C) of 0, and the signals had C values of 0.2, 0.4, 0.6, 0.8 and 1. The standard and signals were generated using values of 5.5 and 1.5 for parameters A and B, respectively. Psychometric functions for parameter A were measured with B and C set to 1.5 and 0.5 respectively. Parameter A was equal to 1 for the standard and to 3.2, 5.5, 7.6, 9.8, 12 for the test signals. Psychometric functions for parameter B were measured with A set to 5.5 and C set to 0.5, the B parameter values used to generate the signals were 3.5 2.1 1.5, 1.2 0.95 0.77. The range of parameter values used for each task are summarised in Table 2. In each condition there were four blocks of 48 trials. In total, normal-hearing listeners completed four separate test sessions of 1 h. The order of the higher and lower modulation rates within a test session was counterbalanced across listeners.

#### 4.1.2 Cochlear implant users

Four adult users of the Med-El cochlear implant took part; all utilized fine structure processing (FSP) speech coding strategies. Spectral input ranges for each of the 12 electrodes, listed in Table 3, were similar across listeners. Implant users completed a 2-h session for two parameters (A and *f*_*0*_).

The method of stimulus generation employed for the CI users was identical to that for the NH listeners, except that, for the discrimination of changes in A, larger differences between the standard and signal stimuli were employed (Table 4). This was done because pilot experiments obtained with the same parameter values as used for the NH listeners resulted in poor performance. The procedure was similar to that used for the NH listeners with the following exceptions:

Prior to each session and for each listener, the listener's most commonly used map was copied onto a laboratory OPUS II speech processor using a clinical software tool provided by Med-El. (A map describes the frequency-to-electrode allocation and the range of current levels to be presented to each electrode in clinical use). Stimuli were D/A converted with an external sound card (Edirol UA-25). The stimuli were then routed into the implant processor via the direct-audio-input. At the beginning of each session, each listener performed a loudness adjustment, intended to establish a comfortable loudness setting for the stimuli. A stimulus was generated with the largest modulation depth and highest modulation rate parameters that would be played during the session the listener was about to perform. This was then played to the listener repeatedly as the output voltage through the sound card was gradually increased. Maximal comfortable levels were obtained by adjusting the level such that the listener rated it ‘loud’ (point seven on an eleven-point scale), and then gradually decreasing the level until the listener rated it ‘comfortably loud’ (point six). The listener was then played examples of stimuli with the combinations of minimum and maximum modulation depth and modulation rates that would be present during the trials, in each case checking whether the stimulus was comfortable and audible. Listener C2 reported that the maximum-modulation-depth/minimum-modulation-rate condition was uncomfortably loud at this stage. The loudness adjustment procedure was then repeated with this stimulus until comfortable. The other parameter combinations were then checked for audibility and comfort using the same loudness rating approach (all were rated at point six, “comfortably loud”), and the output voltage was maintained at this level.

### 4.2 Results and discussion: normal-hearing listeners

#### 4.2.1 Discrimination of changes in the comodulation across carriers

**Fig. 4:**
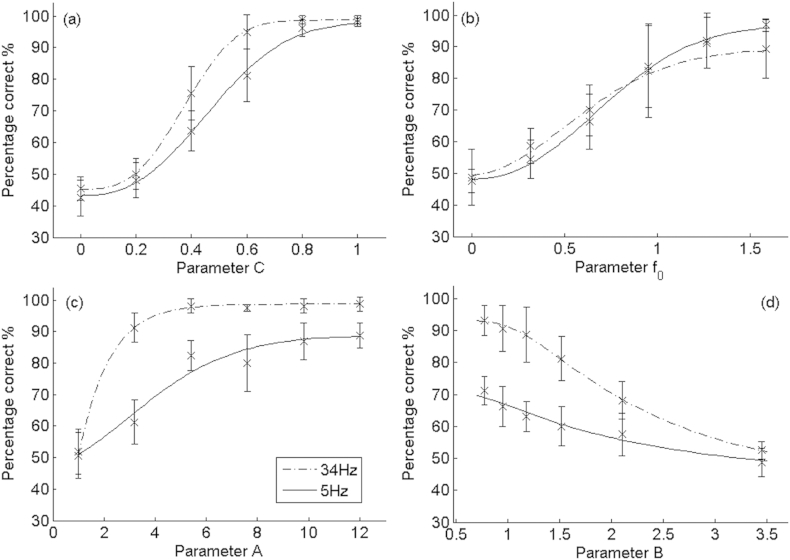
Discrimination of characteristic envelope parameters. Each point shows the group mean performance of the same five NH listeners, with the task performed at two modulation rates (34 Hz and 5 Hz); error bars represent 95% confidence intervals: (a) Discrimination of changes in C (co-modulation) (b) Discrimination of changes in *f*_*0*_ (Rate). (c) Discrimination of changes in A (d) Discrimination of changes in B.

Psychometric functions for the discrimination of the degree of comodulation, averaged across listeners, are shown in Fig. 4a. Performance is defined by the percentage of times an individual correctly selected the interval corresponding to the ‘oddest man out’. Data are shown for the two rates (34 Hz and 5 Hz). In all cases the curves are fitted to the data by a three-parameter modified-Weibull function (Yssaad-Fesselier and Knoblauch, 2006) using a least-squares approach. Error bars indicate 95% confidence intervals calculated from the variation across listeners. Results were similar for the five listeners. A repeated-measures ANOVA was performed with comodulation value and modulation rate as within-subject factors; significance levels reported throughout this article were calculated using the Huynh-Feldt sphericity correction, and the uncorrected degrees of freedom are reported. The ANOVA indicated a highly significant effect of comodulation value (F(5,20) = 113.40; p < 0.01). The effect of modulation rate was not significant (F(1,4) = 4.06; p = 0.11), although the interaction between modulation rate and comodulation value was of borderline significance (F(5,20) = 3.20; p = 0.05). This interaction reflects the fact that performance was better at the higher rate at intermediate, but not at the extreme, values of the comodulation parameter, and so it could be that performance was superior at the higher rate but that this was obscured by floor and ceiling effects. A comodulation value of 0.38 (0.3–0.46 upper and lower 95% confidence limits) for the higher rate and 0.48 (0.38–0.6 upper and lower limits) for the lower rate is required for listeners to achieve 71% correct discrimination, which is the value on which adaptive 2-up 1-down procedures converge (Levitt, 1971). Although we only measured performance with a standard co-modulation value of 0, it can be seen that the psychometric functions are monotonic and that discrimination between other pairs of values would have been possible. For example, the average scores at the slower modulation rate (5 Hz) for comodulation = 0.4 and comodulation = 0.6 were approximately 64% and 83% respectively; for a single listener the 95% confidence intervals (derived from the binomial distribution) for these two values do not overlap. The specific cues used by listeners to detect and process variations in the comodulation parameter are discussed further in Section 6.1.

#### 4.2.2 Discrimination of stochastic modulation rate (*f*_*0*_)

**Fig. 5:**
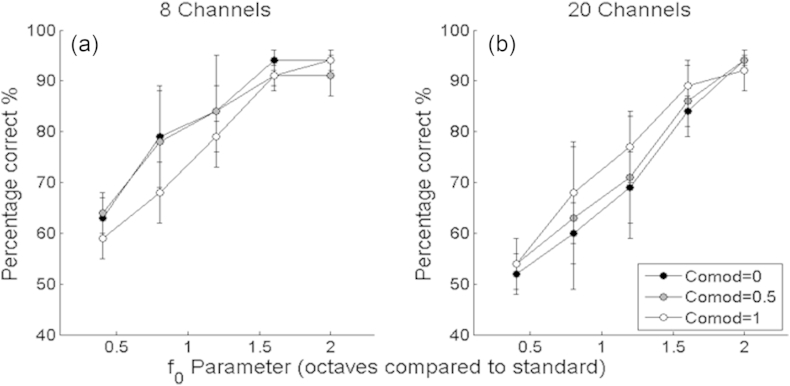
Discrimination of characteristic envelope modulation rate parameter by five normal-hearing listeners. Each point of the psychometric function is averaged across listeners, with co-modulation parameters of 0 (black circles), 0.5 (grey circles) and 1 (unfilled circles). (a) Results for eight-carrier stimuli (b) Results for twenty-carrier stimuli. Error bars represent 95% confidence intervals.

Psychometric functions for discrimination of stochastic modulation rate (*f*_*0*_).

are shown in Fig. 4b. A related-samples ANOVA showed a highly significant effect of test rate (F(5,20) = 45.77; p < 0.01), and confirmed no significant difference between baseline modulation rates (F(1,4) = 0.02; p = 0.969), and with no significant interaction between the rate discrimination and baseline rate (F(5,20) = 0.98; p = 0.45). Although the psychometric functions are monotonic and show that listeners could discriminate differences in envelope rate, performance was considerably poorer than for deterministic envelopes reported previously (Grant et al., 1998; see Section 6.5. for further discussion). To achieve 71% correct, a difference in rate at the input to the generative model of 0.62–0.65 octaves was needed. These values are much higher than the DLs of below 0.1 octaves that have been reported for similar tasks using deterministic stimuli, such as square-wave modulation imposed on noise carriers or sinusoidal modulation of pure-tone carriers (Füllgrabe and Lorenzi, 2003, Grant et al., 1998, Lee, 1994). One possible explanation for this reduced performance is that we measured performance with the comodulation parameter, C, set to 0.5. Because the different bands were not perfectly comodulated, it is possible that the modulation in each band was corrupted by spread of excitation from modulators in neighbouring bands. To assess the influence of altering the relationship between the separate modulators, five different listeners performed a rate discrimination experiment, of the same format as described above, with C set to either 0, 0.5, or 1. This was performed with a slightly lower baseline modulation rate of 24 Hz. The experiment was performed both with eight and with twenty carriers; in both cases there was the same relationship between bandwidth and centre frequency for the carriers i.e. carriers were significantly more widely spaced in the eight-channel condition, reducing the amount of cross-channel “smearing”. The results, shown in Fig. 5, reveal that neither of these manipulations had a marked effect on performance. A repeated measures ANOVA showed no significant effect of the number (8 vs 20) of carriers (F(1,4) = 3.58; p = 0.13), and no significant effect of comodulation value (F(2,8) = 0.03; p = 0.97), with no significant interaction (F(2,8) = 1.997; p = 0.21). Hence we conclude that the rather poor sensitivity to envelope modulation rate observed here must arise from characteristics of the modulator that occur within individual channels, rather than being due to interactions between carriers in the peripheral auditory system. Two likely candidates are that both the position and amplitude of envelope peaks differ from cycle to cycle (Fig.3), and that the modulator varies from presentation to presentation. Unfortunately there is very little data on the rate discrimination of complex but deterministic maskers, and so we cannot be sure whether the poor performance is primarily due to the complexity or stochasticity of our modulators. An exception is the research on the discrimination of second-order modulation (e.g. Füllgrabe and Lorenzi, 2003), which has found rate DLs that are markedly lower than observed here. It is also worth noting that changes in *f*_*0*_ correspond to changes in the upper cutoff of a low-pass modulator, so that, even when two stimuli differ in *f*_*0,*_ both modulation spectra will contain low-frequency components.

#### 4.2.3 Envelope modulation depth (parameters A and B)

**Fig. 6:**
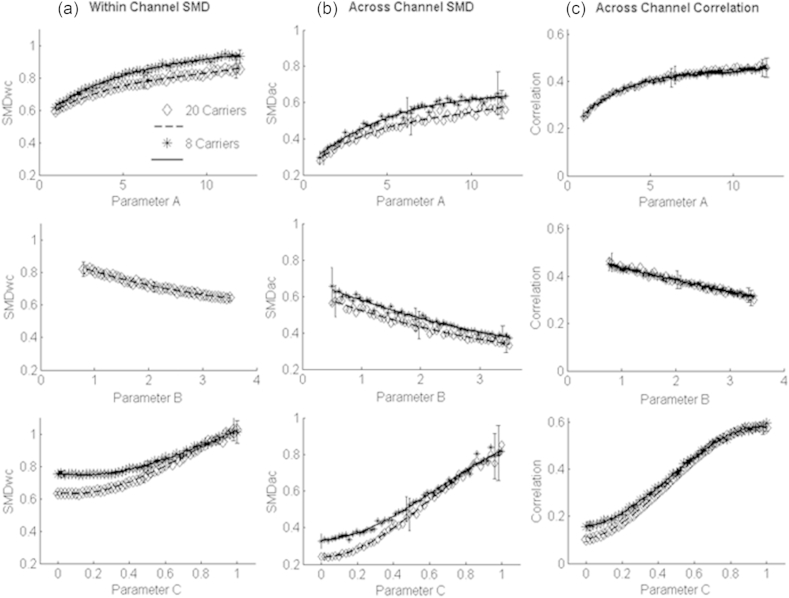
Plots showing envelope statistics as calculated at the output of an auditory model. For each value of the input parameter 32 tokens were generated, with *f*_*0*_ = 34 Hz: Each point shown represents the average of these values. For clarity error bars (+/−1 sd) are shown for an intermediate value of the input parameter and the two extreme values. When one parameter was varied the values of the other parameters were fixed, as in the experiments, at the values shown in Table 2. Broken lines and diamonds show the output when a twenty carrier version of the generative model was used; solid lines and stars indicate results for the eight-carrier condition. From top to bottom, each row shows the effect of manipulating parameters A, B, and C on the three summary statistics which are, from left to right, the within-channel SMD, the across-channel SMD, and the across-channel correlation.

Fig. 4c and d show discrimination performance for the two parameters, A and B, that controlled the modulation depth. In both cases the psychometric functions were monotonic. Unlike the detection of changes in rate, and, possibly, comodulation, performance was markedly better at the higher than at the lower modulation rate. A repeated-measures ANOVA revealed a statistically significant effect of modulation rate – reflecting better performance at the higher rate - for both A (F(1,4) = 65.15; p = 0.01) and B parameters (F(1,4) = 45.76; p < 0.01). There were significant effects of parameters A (F(5,20) = 46.61; p < 0.01) and B (F(5,20) = 29.18; p < 0.01) in the two tasks. The ANOVA also showed significant interactions between modulation rate and both the A (F(5,20) = 7.53; p < 0.01) and B (F(5,20) = 5.74; p < 0.01) parameters. This interaction was due to the presence of floor and/or ceiling effects at the extremes of the psychometric functions. For parameter A, the values corresponding to 71% correct performance were 4.38 and 1.65 for the 5-Hz and 34-Hz rates, respectively. The first two panels in the top row of Fig. 6, which shows our auditory-model simulations based on the 34-Hz modulation rate, illustrates the dependence of the within- and across-channel SMDs on parameter A. It can be seen that the JND of 1.65 corresponds to quite a small difference in SMD between the standard and signal – the mean difference is 0.07 (sd = 0.064) for the within-channel SMD and 0.07 (SD = 0.092) for the across-channel SMD.

### 4.3 Results and discussion: cochlear-implant listeners

#### 4.3.1 Discrimination of stochastic envelope rate (*f*_*0*_)

**Fig. 7:**
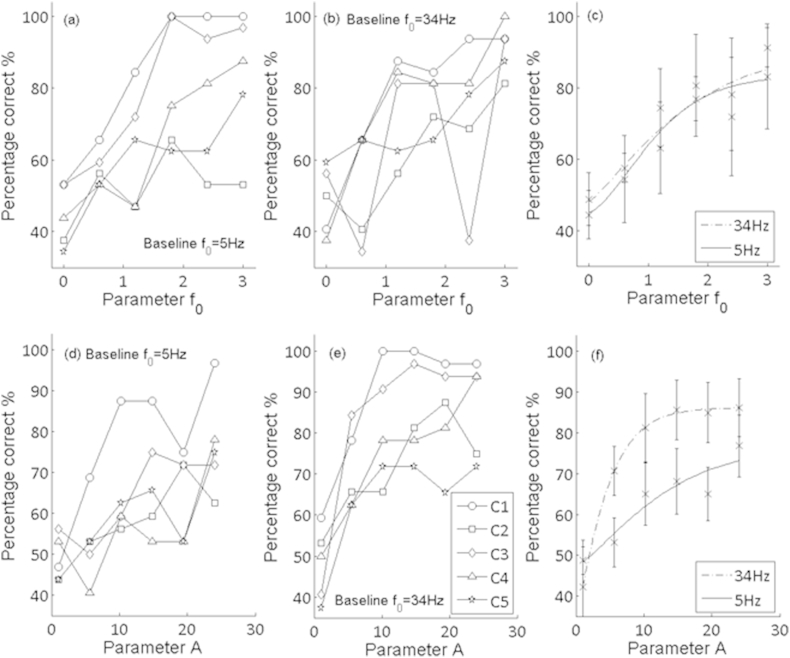
Discrimination of characteristic envelope parameters for five Med-El cochlear implant users. (a) modulation rate discrimination performance at the 5 Hz modulation base-line rate, each line represents performance for a single individual. (b) modulation rate discrimination performance at the 34 Hz modulation base-line rate, each line represents performance for a single individual. (c) each point is the group average performance for the rate discrimination task of the same five CI listeners, with the task performed at two modulation rates (34 Hz and 5 Hz): (d), (e) and (f) show analogous plots for the parameter A, which controlled the modulation depth.

Psychometric functions for discrimination of changes in envelope modulation rate are shown for individual subjects in Fig. 7a and b, and averaged across subjects in Fig. 7c. The variability in performance was greater across the CI users than for the normal-hearing listeners. A repeated-measures ANOVA was performed with the ratio between standard and signal modulation rates, and the modulation rate of the standard (34 Hz vs. 5 Hz) as two within-subject factors. As for the normal-hearing listeners, there was a highly significant effect of rate ratio (F(5,20) = 16.73; p < 0.01) but no significant effect of the standard modulation rate (F(1,4) = 0.22; p = 0.67) and no significant interaction (F(5,20) = 1.02; p = 0.43). Inspection of the average psychometric functions (Fig. 7c) suggests that an alteration in stochastic envelope modulation rate of between 1.5 and 2 octaves is required for listeners to achieve 71% correct discrimination. This is much larger than the discrimination limen (DL) for the normal-hearing listeners of 0.62–0.65 octaves. Performance was also worse than for the discrimination of 20-Hz sinusoidal AM imposed on a high-rate electrical pulse train in the study by Chatterjee and Oberzut (2011); they obtained DLs, averaged across listeners, that were approximately 0.3 and 0.5 octaves in the absence and presence of a level rove, respectively.

#### 4.3.2 Discrimination of changes in parameter A

Psychometric functions for the discrimination of parameter A by implant users are generally monotonic (Fig. 7 lower panels). As for the normal-hearing listeners, discrimination performance is significantly poorer at the lower rate (F(1,4) = 32.65; p < 0.01) with a significant effect of A (F(5,20) = 16.83; p < 0.01), but no significant interaction (F(5, 20); p = 0.139). The change in A required for discrimination at the 71% correct level was larger for the implantees (a value of 6 for the higher rate, compared to 1.65 for the equivalent rate in the normal-hearing listeners). The results for both the *f*_*0*_ and A parameters can be summarised by the statement that CI users performed worse overall than the NH listeners, but that the pattern of results was similar for the two groups.

## 5 Further investigation of the perceptual correlates of Co-modulation in NH listeners

### 5.1 Rationale

The experiment reported in Section 4.2.1 revealed that NH listeners are sensitive to differences in the co-modulation parameter. However this does not mean that listeners were necessarily performing an across-channel comparison between the envelopes at the outputs of separate auditory filters. Rather, because the 20 narrowband noise carriers used to generate our stimuli were contiguous in frequency, it is possible that the comodulation parameter affected the pattern of modulation at the output of each auditory filter. Specifically, it is likely that the modulation depth at the output of an auditory filter that responds to two or more frequency bands will be greater when those bands are modulated by the same, rather than by independent, modulators. This can be seen in the bottom row of Fig. 6, which shows that changes in the comodulation parameter not only affect the cross-channel correlation and the across-channel SMD at the output of the auditory model, but also influence the within-channel SMD.

To investigate further the processing of co-modulation in our stochastic stimuli we performed two additional experiments. The first was simply a re-measurement of psychometric functions for the detection of changes in the A and C parameters for a new set of listeners. Based on the results, three values of A and of C were chosen for each listener so as to generate nine stimuli. These nine stimuli were then compared in a multi-dimensional scaling (MDS) experiment, which had two aims. First, it allowed us to determine whether the two parameters affected the same or different perceptual dimensions. Second, by comparing the results to the outputs of an auditory model, we could gain insight into the cues that listeners were using when making their perceptual judgements. The experiments were carried out with the same 20-channel stimuli as used in the majority of experiments described in Section 4. In order to provide further information on the possible role of interactions between adjacent carriers, the experiments were repeated for two listeners using the eight-channel stimuli (Table 1b, Fig.1b).

### 5.2 Methods

#### 5.2.1 Psychometric functions

Four normal-hearing listeners (S1, S6, S7, S8) took part in the experiments with the 20-channel stimuli; one of these (S1) had previously taken part in the experiments described in Section 4. Listeners S1 and S7 also took part in the experiments with 8-channel stimuli. The modulation rate was 34 Hz. Psychometric functions were measured using the same method as described in that section. For the co-modulation parameter the standard stimulus always had a co-modulation (C) of 0, and the signals had C values of 0.2, 0.4, 0.6, 0.8 and 1. The standard and signals were generated using values of 5.5 and 1.5 for parameters A and B, respectively. Psychometric functions for parameter A were measured with B and C set to 1.5 and 0.5 respectively. Parameter A was equal to 1 for the standard and to 1.6, 2.2, 2.8, 3.4, and 4 for the test signals. For both parameters the psychometric functions were fitted with a three-parameter modified-Weibull function (Yssaad-Fesselier and Knoblauch, 2006) and the just-noticeable difference (JND) was defined as the parameter value corresponding to 71% correct. For each listener three values were then calculated for each parameter, corresponding to that used to generate the standard, 1.5 times the JND, and 3 times the JND, with the constraint that the comodulation parameter was “capped” at a value of one. The three values for each parameter were then combined orthogonally to generate nine stimuli for each listener to be used in the MDS experiment, with fixed values for B (1.5) and *f*_*0*_ (34 Hz).

#### 5.2.2 Multi-dimensional scaling

In each trial of the multi-dimensional scaling task, two 1-s stimuli were presented sequentially using the same equipment as described in Section 4.1.1. The listener was asked to record the perceived difference between the two sounds by using a graphical user interface; moving a sliding bar along a scale that was anchored by labels ‘no difference’ and ‘very different’. 81 stimulus pairs were formed from all possible permutations of the nine stimuli, and each pairing was repeated five times. Thus there were 405 trials in total, and comparisons were made between different stimulus pairs ten times (five times in each of the two possible orders), picked at random without replacement. In this experiment, the stimuli, not just the envelope statistics, were identical across repeats, for a single listener. However, the tokens did differ across listeners.

For each unique combination of stimuli a mean difference rating was obtained from the ten repeats, and this was used to form a dissimilarity matrix. Both metric and non-metric multi-dimensional scaling algorithms (MATLAB statistics toolbox) were then used to create a configuration matrix describing the multi-dimensional scaling solution for one, two, and three dimensions. The metric and non-metric solutions were very similar; only the non-metric solution is shown.

### 5.3 Results

#### 5.3.1 20-band stimuli

The psychometric functions for all listeners and for both parameters were monotonic and led to the JNDs from which the parameter values used in the MDS experiment were calculated (Table 5). These stimuli will be abbreviated using the letters C and A for the co-modulation and A parameters, and the numbers 1, 2, and 3 to describe increasing values of each parameter – for example, stimulus C1A3 was generated using the smallest co-modulation and the largest value of A employed for a given listener. The average JND for parameter C was 0.38, similar to that observed for the listeners tested in the corresponding discrimination experiment described in Section 4. The average JND for the parameter A was 2.43, somewhat higher than the average value of 1.65 obtained for the listeners tested in the corresponding experiment described in Section 4.

**Fig. 8:**
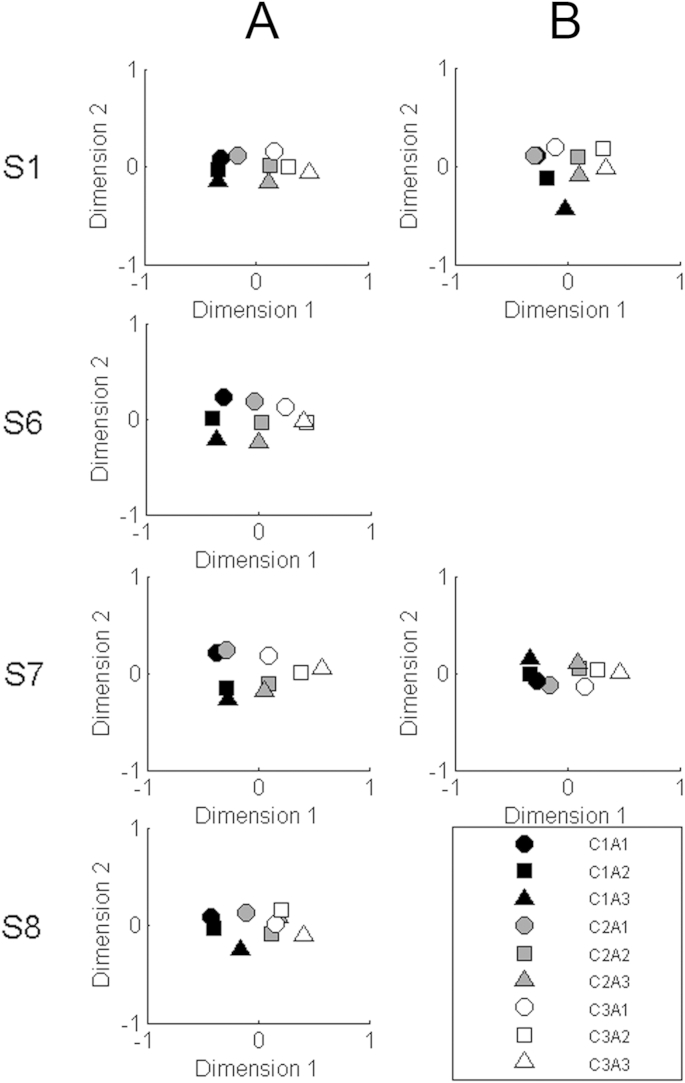
Column A shows non-metric multidimensional scaling (MDS) results (two dimensional solution) for four subjects, for stimuli generated using 20 bandlimited noise carriers. Column B shows the results for two subjects for the eight-carrier stimuli. The exact input values vary between individuals as they reflect 1, 1.5, and 3 times the thresholds obtained in a discrimination task for parameters C and A. Shapes represent different values of parameter A (circle = lowest value of A, square = intermediate value of A, triangle = highest value of A), whilst shading indicates the co-modulation parameter values (black = independent modulators across channels, grey = intermediate co-modulation value, unfilled = highest co-modulation across all channels).

The stress values associated with 1, 2, and 3-dimensional solutions were 0.141, 0.033, and 0.006 respectively. The drop in stress from the one-dimensional to the two-dimensional solution meant that it was possible that the two parameters affected separate perceptual dimensions. Fig. 8 column A therefore shows the results for each listener in a two-dimensional space. It can be seen that the greatest separation is along dimension 1, and that this generally corresponds to differences in the co-modulation value (shading) along that dimension. The value of parameter A (symbol shape) only sometimes has an effect on dimension 1; when this is the case the larger values (triangles) line up in the same direction as increased co-modulation (e.g. subject S7, stimuli C3A1, C3A2, C3A3). Section 6.1 further examines the nature of the dominant perceptual dimension (“dimension 1”) that describes the way in which listeners process co-modulation across channels in stochastic sounds.

There is also some evidence that the A parameter affects a second perceptual dimension; although the points are generally bunched up along dimension 2, the lower values of A (circles) lie at higher points along dimension 2, compared to high values of A (triangles), for all subjects and for all values of the co-modulation parameter. In addition, the intermediate value, A2 (squares), falls between A1 and A3 for eight of the twelve combinations of listener and co-modulation parameter, greater than the four occasions that would be predicted by chance.

#### 5.3.2 8-band stimuli

The JNDs for the 8-band stimuli are shown for parameters C and A in Table 5 for listeners S1 and S7. These values were similar to those obtained for the same listeners with the 20-band stimuli. Because the 20-band stimuli were contiguous whereas the 8-band stimuli were separated by spectral gaps of a minimum of 1.6 semitones between high-frequency carriers and maximum of 2 semitones between low-frequency carriers, this suggests that discrimination of those parameters was not strongly influenced by interactions between adjacent carriers. The MDS results, shown in Fig. 8 column B, also show a pattern similar to that obtained with the 20-channel stimuli. Note that this includes the fact that there was some evidence for parameter A controlling a second dimension; for each listener, the order of the different A parameters (symbol shape) was roughly similar for the three different co-modulation parameters (symbol shading). (One should ignore the fact that high values of A (triangles) produced low dimension 2 scores for listener S1 and high scores for listener S7, because this does not affect the relative distances between points in a two-dimensional space).

## 6 General discussion

### 6.1 Sensitivity to co-modulation in stochastic stimuli

**Fig. 9:**
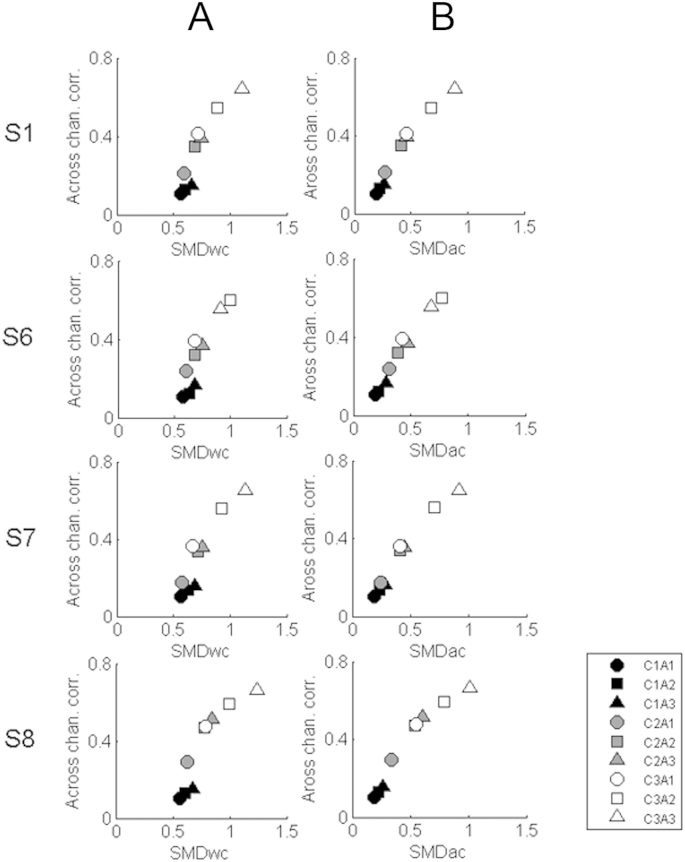
Columns A and B show SMD and cross-channel correlation values determined from the nine stimuli used in the Multi-Dimensional Scaling task, for the four subjects who performed the task with the 20-carrier stimuli. Each row represents the values for a subject. The two columns show results for two different ways of determining the SMD values (column A = within-channel metric, column B = across-channel metric, see text for details).

This section first considers the nature of the perceptual dimension, identified as dimension 1 in our MDS study, that NH listeners primarily used to distinguish the sounds presented in that experiment. Each of the nine stimuli heard by each listener in the MDS experiment was processed using the auditory model described in Section 3. For each stimulus we then calculated the three summary statistics described in that section: the *within-channel SMD*, the *across-channel SMD*, and the *across-channel correlation.* Columns A and B of Fig. 9 show the relationship between both the within-channel and across-channel SMD measures with the across-channel correlation, for the nine stimuli presented to each listener in the MDS experiment.

Fig. 9 column B shows that the across-channel SMD and the cross-correlation measure are highly correlated with each other. The Appendix demonstrates how the two measures are mathematically related, and, in Section 6.2, we will argue that this holds not only for our sounds but for a wide range of broadband stimuli. The correlation between the two measures of course makes it hard for us to determine which one most closely corresponded to the listener's judgement. Although the within-channel SMD (Fig. 9, column A) also co-varies with the cross-correlation, there are some deviations from a simple relationship that can be compared to the MDS results for each listener. Those deviations do not fit the data. For example, for listeners S1, S6, and S7, use of the within-channel cue would predict that stimuli C1A3 (black triangle), C2A2 (grey square) and C3A1 (white circle) would be judged as very similar, whereas in fact C1A3 (black triangle) is well-separated from the other two stimuli on dimension 1 of each listener's MDS results. The MDS results also show that the distance from C3A3 (white triangle) is much greater for C1A3 (black triangle) than for C3A1 (white circle), a fact reflected in both of the across-channel measures (SMD and correlation) but not in the within-channel SMD.

**Fig. 10:**
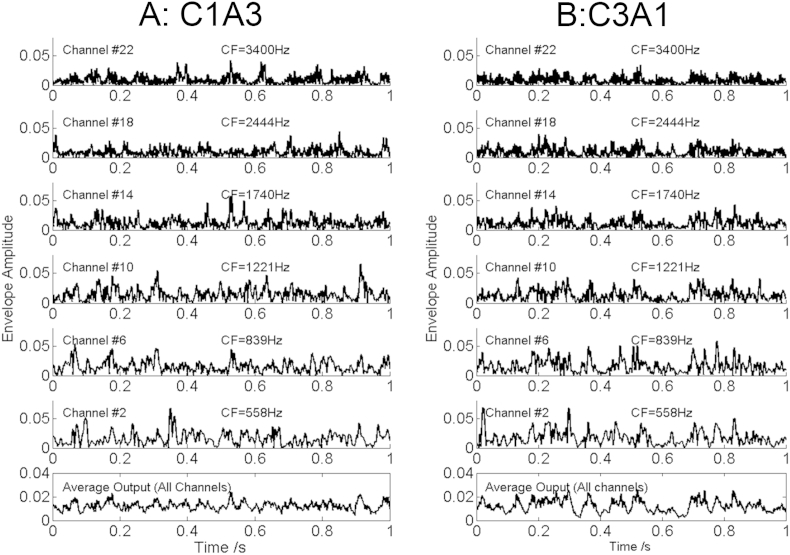
(a) Output envelopes for six channels (channel number and centre frequency shown) of a fourth-order gammatone filterbank for a stimulus with a high value of A and a co-modulation value of zero (stimulus C1A3, as presented to subject S7). (b) As a), but for a stimulus generated using a lower value of A and with the highest co-modulation across frequency channels of the generative model (stimulus C3A1, as presented to subject S7). The bottom row of each column shows the summed envelope (across all channels) for the corresponding stimulus.

In order to compare the correspondence between each of the auditory model parameters with the results of the MDS study, we entered the position of each stimulus on dimension 1 for each listener's MDS as the dependent variable into a univariate ANOVA, with listener as a fixed factor. We then calculated the proportion of the variance accounted for by, separately, the within-channel SMD, the across-channel SMD, and the across-channel correlation. These values, of 71%, 86%, and 95% respectively, all differed significantly from each other, as assessed using Williams' test (p < 0.001 in each case); similar results (not shown) were obtained when the auditory model outputs were analysed using the logarithm of the envelope (cf. Stone and Moore, 2007). We should note that the exact value will depend somewhat on the assumptions made during their calculation. For example, listeners might not correlate every auditory filter output with every other and combine them in a linear way, and so it is possible that even though our across-channel metric does a good job of fitting the data it is not something that the listener can or would compute. However, what does seem clear is that use of the within-channel SMD cue predicts that some different-sounding stimuli should sound similar, at least in terms of the primary perceptual dimension revealed by our MDS experiment. This is illustrated further in Fig. 10, which shows the envelopes for a subset of auditory-filter outputs in response to stimuli C1A3 (equivalent to the black triangle in Fig. 8) and C3A1 (equivalent to the white circle in Fig. 8) for listener S7. The within-channel SMD is similar for the two stimuli (0.70 and 0.68, respectively) but the across-channel sum (bottom row) clearly shows that C3A1 is more modulated, and it indeed does sound more modulated. Example sound stimuli are available online: http://www.mrc-cbu.cam.ac.uk/wp-content/uploads/2015/11/Fig-10-sound-files.zip.

Although, as noted in Section 5.3.1, the ordering of stimuli along dimension 2 of the MDS was not random, and corresponded somewhat with the value of the A parameter of the generative model, none of the correlations between dimension 2 and the within-channel SMD, across-channel SMD, or across-channel correlation were statistically significant.

### 6.2 Relationship between decision metrics and implications for previous studies

**Fig. 11:**
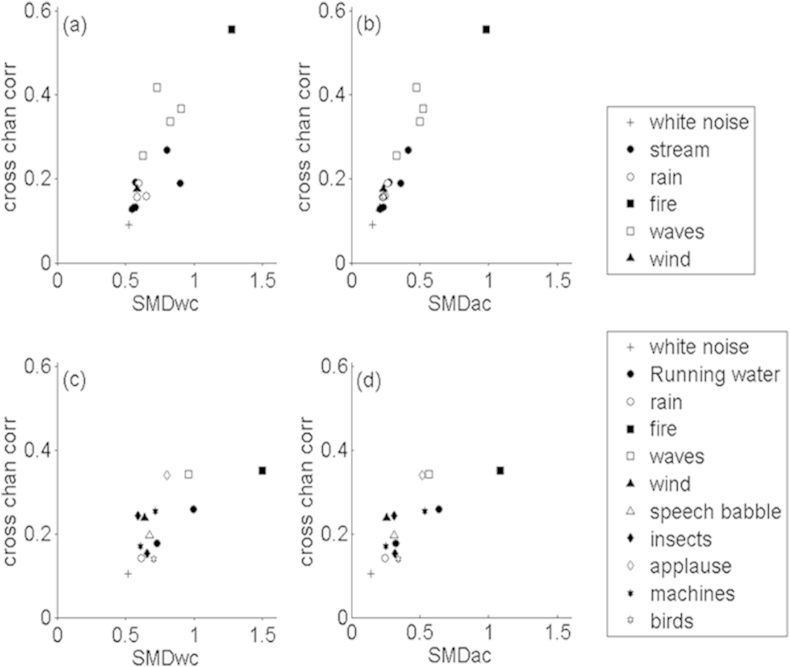
Calculated cross-channel correlation (y-axis) and SMD (x-axis) for a number of environmental sounds (a,b) and for sounds generated by the model described by McDermott and Simoncelli (2011; parts c,d). Parts a and c show the values of SMD calculated using the ‘within-channel’ metric, whilst parts b and d shows SMD calculated using the ‘across-channel’ metric (see text for further details).

The analyses performed so far indicate that the perception of stimulus changes controlled by the co-modulation parameter does involve some genuine across-channel processing, but that we cannot distinguish between the across-channel SMD and the calculation of between-channel correlations. We should stress that this ambiguity is not specific to our stimuli. Fig. 11a and b show the within-channel and across-channel SMD plotted against the cross-channel correlation for a selection of fifteen environmental sounds. The signals were taken from commercially available high-quality sound effects CDs with a sampling rate of 44,100 Hz. The statistics were computed from segments that were 3 s in duration and taken from the steady state of the textures, therefore avoiding artefacts caused by transients. A similar analysis is shown in Fig. 10c and d for a selection of sound textures generated by McDermott and Simoncelli's (2011) model, taken from: http://mcdermottlab.mit.edu/texture_examples/index.html. As can be seen, the across-channel SMD and the cross-correlation co-vary strongly both across the natural and synthesised sounds. Indeed, in the Appendix we show that, provided that the variance and the mean of the envelope is constant across channels, the across-channel SMD is equal to the within-channel SMD multiplied by the square root of the cross-channel correlation (equation (A8)). Although these conditions do not hold exactly across the stimuli represented in Fig. 11, that equation nevertheless does a very good job of predicting the across-channel SMD for those stimuli. For the natural and synthetic (McDermott & Simoncelli) stimulus sets, the correlations between predicted and obtained across-channel SMDs were 0.95 and 0.97 respectively. The average absolute difference between the predicted and obtained values was only 0.01 for the natural stimuli and 0.06 for the synthetic stimuli.

Although differences in the pattern of modulation across frequency bands are sometimes discussed in terms of auditory grouping (e.g. McFadden, 1987, Yost and Sheft, 1989), it is clear that co-modulation can affect the perceptual quality of single perceptual objects, including many environmental sounds and the approximations thereof studied here and elsewhere (McDermott and Simoncelli, 2011, McDermott, 2009, McDermott et al., 2013, Turner and Sahani, 2010). This raises the interesting question of which stimulus parameters determine whether envelope decorrelation leads to sound segregation on the one hand, or, on the other hand, to changes in the quality of a single sound.

Perhaps the strongest cue to sound segregation comes from differences in the onset time between well-separated frequency components (e.g., Darwin and Ciocca, 1992). These onset differences can be viewed as a particularly potent form of envelope decorrelation. There is also some evidence for the effects of decorrelation between the “ongoing” portions of sounds on grouping; for example, a small “pitch pulse asynchrony” between two pulse trains, each filtered into different frequency regions, can lead to the perception of two separate objects (Carlyon, 1994). Two differences between those stimuli and the ones used here are that each pulse in a pulse train has a very steep envelope, and that the pulse trains used by Carlyon (1994) were periodic, whereas our stimuli were modulated by a lowpass noise. Another important feature is, we suspect, that segregation will be more likely when there are two groups of frequency channels, and where the envelope correlation is large within but not between groups. This is one example of how studies that involve a small number of frequency channels may involve perceptual cues that are different from those that are important for sound texture perception. This presence of two groups of frequency channels may not, however, be *sufficient* for perceptual segregation to occur. For example, in studies that require listeners to discriminate changes in correlation between the envelopes of pairs of noise bands (Moore and Emmerich, 1990, Richards, 1987), listeners might discriminate between changes in the across-channel SMD of a single auditory object, rather than perceptually segregating the two bands when they are decorrelated. It is worth noting that, in such studies, an additional noise band is sometimes interspersed between the two target bands, in order to mask within-channel cues. This additional band might impair performance by adding an additional source of variability to the across-channel SMD. A similar explanation might apply to the so-called comodulation detection difference (CDD: Cohen and Schubert, 1987, McFadden, 1987), in which the detection threshold for a narrowband noise target, in the presence of a concurrent group of coherently modulated maskers, is higher when the target is correlated with the maskers than when it is uncorrelated. The decorrelated band might reduce the across-channel SMD, thereby providing an additional cue for detection. It is also worth noting that our conclusion that listeners sum the envelopes across channels is consistent with the phenomenon of Modulation Detection Interference(Yost and Sheft, 1989), whereby the detection of modulation on one carrier is impaired by the presence of irrelevant modulation applied to another carrier that occupies a different frequency region. In the case of MDI, this across-channel summation would impair the independent processing of modulation in different regions; for our paradigm the modulations are combined across channels and form the basis for the listener's discrimination.

We have argued that the across-channel SMD and/or the cross-channel correlation, calculated across the entire bandwidth of the stimulus, accounts for many of the perceptual differences observed in our MDS experiment. However, it is also possible that listeners are sensitive to differences in correlation between two broad frequency channels – for example being co-modulated in the high region and uncorrelated in the low region, compared to *vice versa.* Indeed, McDermott and Simoncelli (2011) reported an improvement in listeners' identification of synthesised sounds when the across-channel correlation was allowed to vary between frequency regions. The mathematical relationship between across-channel SMD and cross-correlation does mean, though, that it is possible that listeners' sensitivity to this feature may have been mediated by the calculation of an across-channel SMD within each of two or more broad frequency regions.

One other feature of the natural-sound analysis shown in Fig. 11 is that the range of the correlation and across-channel SMD values is roughly similar to that covered by our stimuli (cf. Fig. 6). In particular, the largest correlation value in both sets of sounds was about 0.5–0.6. For the natural sounds this value occurred for the sound of a crackling fire. For our experimental stimuli it arose when the input value of the co-modulation parameter equalled 1. In both cases, the maximum correlation at the output of a bank of auditory filters can be limited by the random fluctuations that occur in different frequency regions, which, in the case of the model used here, arose from the stochastic nature of the narrowband carriers. This limitation seems to be similar for “real” and synthesised sound textures. When we replaced the sound textures with a 40-Hz click train, the across-channel correlation increased to 0.87. The fact that this correlation was less than one is likely due to differences in the ringing time of different auditory filters.

### 6.3 Possible role of loudness cues

The stimuli used in our experiments were presented to NH listeners at the same overall rms level, but were not loudness balanced. Because loudness can vary as a function of modulation rate and depth, even for sounds with the same rms, it is theoretically possible that listeners' judgements were influenced by loudness differences between the stimuli. We think that there are several reasons why this is unlikely. First, although loudness can vary with modulation rate, the largest effects occur for narrowband carriers where fast modulation rates lead to side-bands becoming resolved by the auditory system, effectively broadening the excitation pattern (Glasberg and Moore, 2002). The broader frequency spectrum associated with fast modulations effectively spreads the energy into a wider range of auditory filters, thereby reducing the level in each channel and reducing the effect of cochlear compression. However, the sounds used here all had a similar, broadband spectrum. Second, the modulation depth at the output of each auditory filter can affect loudness, either because more peaked filter outputs are more subject to compression, or because listeners selectively weight the higher-amplitude portions of the stimulus. Such effects have been primarily observed when a large number of discrete partials interact within each filter, and where the differences in crest factor – such as between a cosine-phase and a negative-Schroeder-phase harmonic complex – are much greater than observed here (Carlyon and Datta, 1997, Gockel et al., 2003).

In order to assess the possible role of loudness cues, we passed the stimuli used in our 20-channel MDS experiment through the time-varying loudness model described by Glasberg and Moore (2002). That model incorporates the effects of level-dependent auditory filtering and cochlear compression, and successfully accounts for the effects of bandwidth, modulation rate, and duration on loudness. Recall that, for each listener, the stimuli consist of nine combinations of the A and C parameters, each ranging from the value used for the standard in the discrimination experiment up to three times the JND. The largest difference in long-term loudness level across these nine stimuli ranged from 1 phon for listener S6 to 1.5 phons for listener S8. One phon corresponds roughly to a change in signal level of about 1 dB (depending on overall level and frequency region), and so would be barely detectable even for steady broadband sounds; it is likely that such overall level differences would be even less detectable for the stochastically modulated sounds used here. These differences were no larger than those observed when we analysed different exemplars of a sound with the same statistics (using the parameters employed for listener S8, stimulus C3A1). Furthermore, the predicted loudness differences did not always correspond to the perceptual differences observed in our MDS experiment. On average, the softest sound was C1A1 and the loudest was C2A3. Although C1A1 tends to lie towards the lowest end of dimension 1 in the MDS results, C2A3 tends to lie in the middle. Hence we conclude that it is very unlikely that listeners' judgements were mediated by differences in long-term loudness. Of course, there may well have been a difference in the extent to which loudness varied *within* each stimulus, because, as we have shown, the across-channel SMD differed reliably between stimuli. This would be consistent with our argument that differences in the distribution of signal energy over time are of one of the cues that listeners use to distinguish between sound textures.

We were unable to perform a similar analysis for the CI experiments, both because we know of no well-established model that accounts for the loudness of modulated sounds in electric hearing, and because our CI listeners did not perform a multidimensional scaling experiment. However, it is worth noting that the loudnesses of all of the stimuli presented to the CI listeners were judged to correspond to the same point on an eleven-point rating scale. Additional evidence that CI listeners were not using loudness cues comes from the observation that detection of differences in the A and ***f***_***0***_ parameters had a similar dependence on baseline ***f***_***0***_ as for the NH listeners, with detection of differences in A, but not ***f***_***0***_, being better at the higher overall ***f***_***0***_ in both groups. The most parsimonious explanation for this similarity is that the two groups were using similar cues. Hence although we cannot completely rule out the use of some loudness cues by CI listeners, we believe it unlikely that they had a major contribution on the results reported here.

### 6.4 Statistical modulation depth, skew, and kurtosis

So far, we have discussed the perceptual effects of changes in parameters A and B in terms of changes in the SMD. However, these parameters also influence the skew and kurtosis of the envelopes. Generally, the perceptual effects of these higher-order envelope moments have proved difficult to distinguish from those of parameters that correspond to changes in the modulation depth. For example, Lorenzi et al. (1999, 1999) required NH listeners to detect phase differences between two sinusoidal modulators applied to a white-noise carrier, and measured performance as a function of the modulation depth of the lower-rate modulator. They implemented a model consisting of a 2-kHz wide bandpass filter centred on 5 kHz, followed by half-wave rectification, low-pass filtering, and a decision device. They found that performance could be predicted reasonably well when the decision was based on either the skew or the crest factor, but they could not determine which of these two metrics best accounted for the data. Strickland and Viemeister (1996) used a similar approach to determine the envelope statistic that best predicted performance on the detection of a target modulation, in the presence of a masker modulator, as a function of the signal modulation rate. They found that performance was best captured by the ratio of the maximum and minimum envelope amplitudes.

We implemented a modified version of our auditory filter model (Section 3) to determine the envelope statistics that listeners were likely to be using when detecting changes in parameter A. Two exemplars were created, one with parameters set to those of the standard stimulus used in the experiment and one with the A parameter set to the value required to achieve 71% in the discrimination experiment (see Section 4.2.3)The difference in the across channel SMD was calculated between the two exemplars. This process was then repeated 2000 times, and a mean difference in the across channel SMD was measured, along with the standard deviation of the differences. When expressed as a z-score the value, 0.96 for the across channel SMD, was larger than when the same process was repeated for the envelope skew (z-score = 0.03), and envelope kurtosis (z-score = 0.11).

### 6.5 Effect of modulation rate on the discrimination of envelope statistics

An interesting aspect of the present results is that performance was significantly better at 34 than at 5 Hz for the parameters A and B, which controlled modulation depth, but not for changes in the rate or co-modulation parameters. Here we discuss these findings with reference to the results reported previously in the literature and to the analyses presented elsewhere in this article.

We believe that ours are the first data on rate discrimination obtained with stochastic modulators. Previous data, obtained with deterministic modulators, differ in the effect of baseline rate on sensitivity to changes in modulator rate. Mowbray et al. (1956) measured difference limens for the frequency of a square-wave modulation applied to broadband noise, for modulation frequencies between 0.4 and 320 Hz. They found that the minimum detectable increase in modulation frequency, Δ*f*, increased markedly as the baseline modulator frequency, *f*, increased above 5 Hz. In contrast, both Formby (1985) and Hanna (1992) reported that Δ*f* was roughly constant for modulator frequencies between 20 and 60 Hz. When converted to relative values, as used here, Δ*f/f* in Mowbray *et* al’ s study, calculated from their fit to the data, would have been higher at 34 Hz than at 5 Hz, whereas Δ*f/f* would have decreased with increasing baseline rate in both Formby's and Hanna's experiment.

The results described in Section 4.2 showed a significant effect of baseline rate on discrimination of the A and B parameters, but not for the discrimination of changes in the co-modulation parameter. However, as noted in Section 4.2.1, the absence of a main effect might have been due to ceiling and floor effects; inspection of Fig. 4 reveals that, for four out of the six points on the psychometric function, average performance at the 5-Hz rate was very close to either 50 or 100%. For the two remaining points performance was better by about 12–15% at the higher rate, and, as mentioned in Section 4.2.1, there was a borderline interaction between baseline rate and the co-modulation parameter used for the signal. Hence it is possible that there is an effect of baseline rate on sensitivity to changes in co-modulation. This would be consistent with the findings of Moore and Emmerich (1990), who required listeners to discriminate pairs of noise bands that were correlated from otherwise identical pairs that were uncorrelated. They found that performance was better when the noise bandwidths were 100 Hz than when they were 25 Hz. The modulation spectrum of a narrowband noise has a lowpass characteristic, with a cut-off that is proportional to the noise bandwidth. They interpreted the better correlation discrimination performance with a 100-Hz than with a 25-Hz-wide bandwidth in terms of the increase in the number of envelope fluctuations at the wider bandwidth. This interpretation was bolstered by the fact that performance at the narrower bandwidth with a duration of 500 ms was as good as that at the wider bandwidth at a 100-ms duration. Their results suggest that the processing of across-frequency correlation should be better for higher modulator cut-off frequencies, although it should be noted that the cut-off frequencies used in their study were higher than the lowest value of 5 Hz studied here.

It is also possible that the stochasticity of our stimuli was responsible for the effect of baseline rate on the detection of changes in the A and B parameters. For deterministic modulators, the detection of differences in modulation depth is independent of frequency over the range between 5 and 34 Hz, at least for conditions where the AM in the standard interval is above threshold (Ozimek and Sek, 1988, Wakefield and Viemeister, 1990). For stochastic modulators such as ours, the modulation depth between adjacent peaks and valleys in the stimulus will have varied from cycle to cycle of the modulator. If sensitivity (d′) is limited by this external variance, it should increase with the square root of the number of cycles of modulation. In contrast, for deterministic modulators, performance may be limited by variance inherent to the listener's encoding of the stimulus. If some of that variance occurs after combination of information across cycles then we might expect a smaller effect of baseline modulation rate for deterministic modulators.

## 7 Implications for the study of sound textures by CI users

Section 1 described the possible application of our generative model to the study of the perception of stochastic stimuli by CI users, and for the development of a method by which a CI patient with tinnitus might select an appropriate tinnitus relieving sound. The results described in Section 4.2, 4.3 show that CI users demonstrate a similar qualitative pattern to NH listeners, with discrimination of A but not *f*_*0*_ being better at the higher baseline modulation rate. This is encouraging as it suggests that the mechanisms of perception of sound textures by CI users might be successfully studied with NH listeners, in the same way as studies using noise vocoders - a technique not dissimilar from that used here – have informed our understanding of speech perception by CI users (Shannon et al., 1995). A *caveat* is that the overall level of performance was lower for CI than for NH listeners. Further validation of this idea will require experiments using supra-threshold tasks, such as the MDS experiment reported here for NH listeners. Such experiments are currently underway in our laboratory.

## 8 Summary

i) We have determined the sensitivity of NH and CI listeners to parameters of a stochastic sound texture generator.
ii) Discrimination by NH listeners of changes in two model parameters that controlled the modulation depth in each channel was better at higher overall modulation rates. However no effect of overall rate was observed for the detection of changes in modulation rate.
iii) Rate discrimination by NH listeners was significantly poorer for our complex stochastic modulation envelopes than has been reported previously for the discrimination of sinusoidal AM. This difference is not due to smearing across auditory filters and/or due to non-perfect co-modulation across channels.
iv) NH listeners are sensitive to the degree of co-modulation across multiple modulated band-limited noise carriers. Our analysis suggests that this is unlikely to be due to the detection of differences in the average within-channel modulation depth, and may reflect sensitivity to the overall modulation depth of the stimulus, derived by summing the outputs across multiple auditory filters.
v) The pattern of results observed for CI listeners was generally similar to that for NH listeners. They also showed better discrimination of changes in a parameter that controlled modulation depth at higher overall modulation rates. As for NH listeners, no effect of overall rate was observed for the detection of changes in modulation rate. Performance was, however, worse overall for CI users than for NH listeners.

## Figures and Tables

**Table 1:** Centre frequencies, equivalent rectangular bandwidths (ERBs) and Q-values (CF/ERB, for the ERBs shown) for a) the twenty carrier version and b) the eight-carrier version of the generative model.

Centre frequency, Hz	ERB, Hz	Q (ERB)
a)		
499	122	4.1
571	134	4.3
648	147	4.4
733	162	4.5
827	177	4.7
928	194	4.8
1042	213	4.9
1165	235	5
1299	257	5.1
1448	282	5.1
1610	310	5.2
1787	340	5.3
1984	373	5.3
2199	409	5.4
2434	449	5.4
2691	492	5.5
2975	540	5.5
3285	593	5.5
3626	650	5.6
4000	713	5.6
b)		
499	122	4.1
709	158	4.5
976	203	4.8
1320	261	5.1
1762	335	5.3
2330	431	5.4
3061	554	5.5
4000	713	5.6

**Table 2:** Parameter values at the input to the generative model (normal-hearing listeners). See text for specific values (Section 4.1.1).

Input Parameter	Condition			
B	A	*f*_*0*_	C
B	**0.8–3.5**	1.5	1.5	1.5
A	5.5	**1**–**12**	5.5	5.5
*f*_*0*_	5 Hz or 34 Hz	5 Hz or 34 Hz	**5**–**15 Hz or 34–103 Hz**	5 Hz or 34 Hz
C	0.5	0.5	0.5	**0**–**1**

The figures in bold represent the range for the input variable.

**Table 3:** Centre frequencies for each of the channels corresponding to the 12 electrodes of the Med-El cochlear implant device.

Electrode number	Centre frequency, Hz
1	149
2	262
3	409
4	602
5	852
6	1183
7	1632
8	2280
9	3064
10	4085
11	5656
12	7352

**Table 4:** Parameter values at input to generative model (CI users).

Input Parameter	A	*f*_*0*_
B	1.5	1.5
A	**1**–**24**	**5.5**
*f*_*0*_	5 Hz or 34 Hz	**5**–**15 Hz or 34–103 Hz**
C	0.5	0.5

**Table 5:** Discrimination thresholds obtained from discrimination tasks performed for stimuli with eight or twenty narrowband noise carriers. The discrimination task was performed immediately prior to the multidimensional scaling task; input parameters for the MDS task were chosen to correspond to the standard stimuli and to be 1.5 and 3 times these JNDs, with the constraint that the comodulation parameter could not exceed one.

Subject	Discrimination thresholds (71% correct)			
8 Channels		20 Channels	
C	A	C	A
S1	0.3	2.1	0.3	2
S6			0.3	1.9
S7	0.4	2	0.3	3.5
S8			0.4	2.3

## Appendix

This appendix shows how the modulation depth of the cross-channel envelope mathematically relates to the between-channel cross-correlations.

Let *a*_*dt*_ represent the envelope in band *d* = 1,2…*D* at time *t* = 1,2…*T*. In what follows, averages over time are denoted using the short hand, ${\langle a_{dt}\rangle }_{t}=\frac{1}{T}\sum_{t=1}^{T}a_{dt}$, which is the average value of the envelope in each channel. Similarly, averages over channels are denoted ${\langle a_{dt}\rangle }_{d}=\frac{1}{D}\sum_{d=1}^{D}a_{dt}$ , which is called the cross-channel envelope. Averages over both time and channels are denoted ${\langle a_{dt}\rangle }_{dt}=\frac{1}{DT}\sum_{d=1,t=1}^{D,T}a_{dt}$ , which is the average value of the cross-channel envelope.

Using this notation, the covariance between envelopes in two different channels, denoted *d* and *d′* is defined as:

(A1) $$cov_{dd^{\prime }}={\langle a_{dt}a_{d^{\prime }t}\rangle }_{t}-{\langle a_{dt}\rangle }_{t}{\langle a_{d^{\prime }t}\rangle }_{t}$$

Then the variance of the cross-channel envelope,

(A2) $$var\left({\langle a_{dt}\rangle }_{d}\right)={\langle {\langle a_{dt}\rangle }_{d}^{2}\rangle }_{t}-{\langle {\langle a_{dt}\rangle }_{d}\rangle }_{t}^{2}=\frac{1}{D^{2}}\sum_{d=1,d^{\prime }=1}^{D,D}{\langle a_{dt}a_{d^{\prime }t}\rangle }_{t}-\frac{1}{D^{2}}\sum_{d=1,d^{\prime }=1}^{D,D}{\langle a_{dt}\rangle }_{t}{\langle a_{d^{\prime }t}\rangle }_{t}=\frac{1}{D^{2}}\sum_{d=1,d^{'}=1}^{D,D}\left[\left({\langle a_{dt}a_{d^{\prime }t}\rangle }_{t}-{\langle a_{dt}\rangle }_{t}{\langle a_{d^{\prime }t}\rangle }_{t}\right)\right]=\frac{1}{D^{2}}\sum_{d=1,d^{\prime }=1}^{D,D}cov_{dd^{\prime }}={\langle cov_{dd^{\prime }}\rangle }_{dd^{\prime }}$$

In other words, the variance of the cross-channel envelope is equal to the average covariance between the envelope amplitudes in different channels.

The statistical modulation depth of the across-channel envelope is defined as.

(A3) $$\;SMD\left({\langle a_{dt}\rangle }_{d}\right)=\frac{\sqrt{var\left({\langle a_{dt}\rangle }_{d}\right)}}{{\langle a_{dt}\rangle }_{dt}}=\frac{\sqrt{{\langle cov_{dd^{\prime }}\rangle }_{dd^{\prime }}}}{{\langle a_{dt}\rangle }_{dt}}\;\;\;$$

The correlation between two channels is the covariance normalised by the square root of the product of the associated variances:

(A4) $$cor_{dd^{\prime }}=\frac{cov_{dd^{\prime }}}{\sqrt{cov_{dd}cov_{d^{\prime }d^{\prime }}}}$$

Hence the across-channel SMD is given by:

(A5) $$SMD\left({\langle a_{dt}\rangle }_{d}\right)=\frac{\surd \left({\langle cor_{dd^{\prime }}\sqrt{cov_{dd}cov_{d^{\prime }d^{\prime }}}\;\rangle }_{dd^{\prime }}\;\right)}{{\langle a_{dt}\rangle }_{dt}}$$

If (condition 1) the variance of the envelope in each channel is constant $cov_{dd}=c_{1}$, then A5 can be rewritten as:

(A6) $$SMD\left({\langle a_{dt}\rangle }_{d}\right)=\left(\sqrt{c_{1}}/{\langle a_{dt}\rangle }_{dt}\right)\sqrt{{\langle cor_{dd^{\prime }}\rangle }_{d,d^{\prime }}}$$

The within-channel SMD is defined as the within-channel standard deviation divided by the within-channel mean, averaged across all channels. If condition 1 holds, the within-channel SMD can be expressed as:

(A7) $${\langle SMD\left(a_{dt}\right)\rangle }_{d}={\langle \left(\surd c_{1}/{\langle a_{dt}\rangle }_{t}\right)\rangle }_{d}$$

If, additionally, the within-channel mean ${\langle a_{dt}\rangle }_{t}$ is constant across channels (condition 2), then (by combining A6 and A7) the across-channel SMD is proportional to the within-channel SMD multiplied by the square root of the average across-channel correlation:

(A8) $$SMD\left({\langle a_{dt}\rangle }_{d}\right)={\langle SMD\left(a_{dt}\right)\rangle }_{d}\sqrt{{\langle cor_{dd^{\prime }}\rangle }_{d,d^{\prime }}}$$

## References

[bib1] Carlyon R.P. (1994). Detecting pitch-pulse asynchronies and differences in fundamental frequency. J. Acoust. Soc. Am..

[bib2] Carlyon R.P., Datta a.J. (1997). Masking period patterns of Schroeder-phase complexes: effects of level, number of components, and phase of flanking components. J. Acoust. Soc. Am..

[bib3] Carlyon R.P., Macherey O., Frijns J.H.M., Axon P.R., Kalkman R.K., Boyle P., Baguley D.M., Briggs J., Deeks J.M., Briaire J.J., Barreau X., Dauman R. (2010). Pitch comparisons between electrical stimulation of a cochlear implant and acoustic stimuli presented to a normal-hearing contralateral ear. J. Assoc. Res. Otolaryngol..

[bib4] Cazals Y., Pelizzone M., Saudan O., Boex C. (1994). Low-pass filtering in amplitude modulation detection associated with vowel and consonant identification in subjects with cochlear implants. J. Acoust. Soc. Am..

[bib5] Chatterjee M. (2003). Modulation masking in cochlear implant listeners: envelope versus tonotopic components. J. Acoust. Soc. Am..

[bib6] Chatterjee M., Oba S.I. (2004). Across- and within-channel envelope interactions in cochlear implant listeners. J. Assoc. Res. Otolaryngol..

[bib7] Chatterjee M., Oberzut C. (2011). Detection and rate discrimination of amplitude modulation in electrical hearing. J. Acoust. Soc. Am..

[bib8] Chatterjee M., Robert M.E. (2001). Noise enhances modulation sensitivity in cochlear implant listeners: stochastic resonance in a prosthetic sensory system?. JARO - J. Assoc. Res. Otolaryngol..

[bib9] Cohen M.F., Schubert E.D. (1987). The effect of cross-spectrum correlation on the detectability of a noise band. J. Acoust. Soc. Am..

[bib10] Cooke M.P. (1991). Modelling Auditory Processing and Organisation.

[bib11] Darwin C.J., Ciocca V. (1992). Grouping in pitch perception: effects of onset asynchrony and ear of presentation of a mistuned component. J. Acoust. Soc. Am..

[bib12] Formby C. (1985). Differential sensitivity to tonal frequency and to the rate of amplitude modulation of broadband noise by normally hearing listeners. J. Acoust. Soc. Am..

[bib13] Fu Q.J. (2002). Temporal processing and speech recognition in cochlear implant users. Neuroreport.

[bib14] Füllgrabe C., Lorenzi C. (2003). The role of envelope beat cues in the detection and discrimination of second-order amplitude modulation. J. Acoust. Soc. Am..

[bib15] Glasberg B.R., Moore B.C.J. (2002). A model of loudness applicable to time-varying sounds. J. Audio Eng. Soc..

[bib16] Gockel H., Moore B.C.J., Patterson R.D., Meddis R. (2003). Louder sounds can produce less forward masking: effects of component phase in complex tones. J. Acoust. Soc. Am..

[bib17] Grant K.W., Summers V., Leek M.R. (1998). Modulation rate detection and discrimination by normal-hearing and hearing-impaired listeners. J. Acoust. Soc. Am..

[bib18] Handscomb L. (2006). Use of bedside sound generators by patients with tinnitus-related sleeping difficulty: which sounds are preferred and why?. Acta Otolaryngol. Suppl..

[bib19] Hanna T.E. (1992). Discrimination and identification of modulation rate using a noise carrier. J. Acoust. Soc. Am..

[bib20] Hobson J., Chisholm E., El Refaie A. (2010). Sound therapy (masking) in the management of tinnitus in adults. Cochrane Libr..

[bib21] Lee J. (1994). Amplitude modulation rate discrimination with sinusoidal carriers. J. Acoust. Soc. Am..

[bib22] Levitt H. (1971). Transformed up-down methods in psychoacoustics. J. Acoust. Soc. Am..

[bib23] Lorenzi C., Berthommier F., Demany L. (1999). Discrimination of amplitude-modulation phase spectrum. J. Acoust. Soc. Am..

[bib24] Lorenzi C., Gallego S., Patterson R.D. (1998). Amplitude compression in cochlear implants artificially restricts the perception of temporal asymmetry. Br. J. Audiol..

[bib25] Lorenzi C., Gallégo S., Patterson R.D. (1997). Discrimination of temporal asymmetry in cochlear implantees. J. Acoust. Soc. Am..

[bib26] McDermott H.J., McKay C.M., Vandali A.E. (1992). A new portable sound processor for the university of Melbourne/nucleus limited multielectrode cochlear implant. J. Acoust. Soc. Am..

[bib27] McDermott J.H. (2009). The cocktail party problem. Curr. Biol..

[bib28] McDermott J.H., Schemitsch M., Simoncelli E.P. (2013). Summary statistics in auditory perception. Nat. Neurosci..

[bib29] McDermott J.H., Simoncelli E.P. (2011). Sound texture perception via statistics of the auditory periphery: evidence from sound synthesis. Neuron.

[bib30] McFadden D. (1987). Comodulation detection differences using noise-band signals. J. Acoust. Soc. Am..

[bib31] Moore B.C., Emmerich D.S. (1990). Monaural envelope correlation perception, revisited: effects of bandwidth, frequency separation, duration, and relative level of the noise bands. J. Acoust. Soc. Am..

[bib32] Mowbray G.H., Gebhard J.W., Byham C.L. (1956). Sensitivity to changes in the interruption rate of white noise. J. Acoust. Soc. Am..

[bib33] Ozimek E., Sek A. (1988). AM difference limens for noise bands. Acta Acust..

[bib34] Richards V.M. (1987). Monaural envelope correlation perception. J. Acoust. Soc. Am..

[bib35] Richardson L.M., Busby P.A., Clark G.M. (1998). Modulation detection interference in cochlear implant subjects. J. Acoust. Soc. Am..

[bib36] Shannon R.V. (1992). Temporal modulation transfer functions in patients with cochlear implants. J. Acoust. Soc. Am..

[bib37] Shannon R.V., Zeng F.G., Kamath V., Wygonski J., Ekelid M. (1995). Speech recognition with primarily temporal cues. Science.

[bib38] Stone M.A., Moore B.C.J. (2007). Quantifying the effects of fast-acting compression on the envelope of speech. J. Acoust. Soc. Am..

[bib39] Strickland E.a., Viemeister N.F. (1996). Cues for discrimination of envelopes. J. Acoust. Soc. Am..

[bib40] Turner R.E. (2010). Statistical Models for Natural Sounds.

[bib41] Turner R.E., Sahani M. (2010). Statistical inference for single- and multi-band probabalistic amplitude demodulation. Proceedings of the IEEE International Conference on Acoustics, Speech, and Signal Processing, 2010. Dallas.

[bib42] Wakefield G.H., Viemeister N.F. (1990). Discrimination of modulation depth of sinusoidal amplitude modulation (SAM) noise. J. Acoust. Soc. Am..

[bib43] Wilson B.S., Finley C.C., Lawson D.T., Wolford R.D., Eddington D.K., Rabinowitz W.M. (1991). Better speech recognition with cochlear implants. Nature.

[bib44] Won J.H., Drennan W.R., Nie K., Jameyson E.M., Rubinstein J.T. (2011). Acoustic temporal modulation detection and speech perception in cochlear implant listeners. J. Acoust. Soc. Am..

[bib45] Yost W.A., Sheft S. (1989). Across-critical-band processing of amplitude-modulated tones. J. Acoust. Soc. Am..

[bib46] Yssaad-Fesselier R., Knoblauch K. (2006). Modeling psychometric functions in R. Behav. Res. Methods.

